# The Mediating Role of AI Ethics Awareness Between AI Attitudes and AI Utilization: A Multisite Cross‐Sectional Study of Saudi Nursing Students

**DOI:** 10.1155/jonm/5338801

**Published:** 2026-07-22

**Authors:** Daniel Joseph E. Berdida, Noura Alhudaib, Rizal Angelo N. Grande, Salah A. Alsharafi, Cyrelle D. Agunod, Adelina M. Santos, Hanay Huwaydi Alanazi, Modi Al-Moteri, Nada Alqarawi, Ibrahim Alasqah

**Affiliations:** ^1^ Nursing Department, North Private College of Nursing, Arar, Northern Border, Saudi Arabia; ^2^ Office of the Dean, North Private College of Nursing, Arar, Northern Border, Saudi Arabia; ^3^ Nursing Department, Fakeeh College for Medical Sciences, Jeddah, Makkah, Saudi Arabia, fakeehcollege.edu.sa; ^4^ Medical Sciences and Preparatory Year Department, North Private College of Nursing, Arar, Northern Border, Saudi Arabia; ^5^ Medical Surgical Nursing Department, College of Nursing, Taif University, Taif 21944, Saudi Arabia, tu.edu.sa; ^6^ Department of Community, Psychiatric and Mental Health Nursing, College of Nursing, Qassim University, Buraydah Qassim, 52571, Saudi Arabia, qu.edu.sa; ^7^ Nursing Services Department, Qassim University Medical City, Buraydah 52571, Saudi Arabia; ^8^ School of Health, University of New England, Armidale, New South Wales, 2351, Australia, une.edu.au

**Keywords:** artificial intelligence, attitude, awareness, ethics, nursing, students, technology

## Abstract

**Aims and Objectives:**

To investigate the mediating role of nursing students’ artificial intelligence (AI) ethics awareness between the association of attitudes toward AI technology and perceived AI utilization.

**Background:**

A paucity of studies exists about the role of AI ethics awareness between attitudes toward AI technology and perceived AI utilization, particularly among nursing students.

**Methods:**

A multisite cross‐sectional, correlational research participated by nursing students (*n* = 765) that were consecutively recruited from four nursing colleges (two private and two public owned) in Saudi Arabia. Three standardized self‐report scales were utilized to collect data, and covariance‐based structural equation modeling, using maximum likelihood estimation and bias‐corrected bootstrap method, and mediation analyses were employed for data analyses.

**Results:**

The mean age of participants was 23.98 years old (SD = 4.40), and majority were females (78.90%), were second year students (33.85%), and had a mean grade point average of 4.16 (SD = 0.65). AI technology attitudes were positively associated with AI ethics awareness (*β* = 0.84, *p* = 0.001, and 95% CI = 0.76–0.94) and AI utilization (*β* = 0.55, *p* = 0.010, and 95% CI = 0.13–0.97). AI ethics awareness was positively associated with AI utilization (*β* = 0.41, *p* = 0.043, and95% CI = 0.01–0.84). Mediation analysis showed that AI technology attitudes were indirectly associated with AI utilization (*β* = 0.36, *p* = 0.013, and 95% CI = 0.05–0.68) via the mediation of AI ethics awareness. AI technology attitudes measured 72.05% of the *R* ^2^ of AI ethics awareness, while both AI ethics awareness and AI technology attitudes measured 87.94% of the *R* ^2^ of AI utilization.

**Conclusion:**

Nursing students’ AI technology attitudes and AI ethics awareness were positively associated with AI utilization, whereas AI ethics mediated between AI technology attitudes and AI utilization. Policymakers, nursing educational institutions, and educators could integrate AI ethics into nursing curricula to cultivate positive AI attitudes and responsible AI usage, preparing future nurses for an AI‐integrated healthcare environment.

**Implications for Nursing Management:**

Nurse administrators in the nursing educational institutions and nurse managers in affiliated clinical training centers should provide a conducive learning environment (i.e., adequate resources, teaching/learning materials, and AI‐trained staff) where students can learn AI‐integrated technologies through practical or simulated activities.

## 1. Introduction

Artificial intelligence (AI) is increasingly shaping everyday life and professional practice, including healthcare and higher education. Broadly, AI refers to systems that use data, computational models, and connectivity to perform tasks commonly associated with human intelligence, such as perception, reasoning, learning, problem‐solving, and interaction [1]. Although these capabilities create important opportunities, AI use also raises substantial ethical concerns. These include risks related to bias, privacy, accountability, human rights, patient safety, and the spread of misinformation and disinformation [2, 3]. In response to such concerns, UNESCO issued the Recommendation on the Ethics of Artificial Intelligence in 2021, which emphasizes that AI development and use should be aligned with human rights, ethical standards, and appropriate governance structures [4]. In healthcare, this guidance is especially relevant because AI applications may directly affect clinical decisions, care quality, and public trust.

AI technologies are already transforming healthcare systems through their use in patient care, diagnostics, workflow management, and administrative processes [5]. Nursing practice and nursing education are part of this transformation [6]. In clinical settings, AI tools can support care planning, patient monitoring, documentation, and decision support across the continuum of care [7]. In educational settings, AI has been integrated into nursing curricula through approaches such as intelligent tutoring systems, machine learning–based learning tools, and simulation technologies designed to strengthen students’ knowledge and clinical performance [8, 9]. These developments suggest that future nurses will be expected not only to understand AI technologies but also to use them responsibly and critically within professional practice.

Existing research in nursing education suggests that AI‐supported learning approaches may enhance clinical reasoning, decision‐making, and student engagement [8, 9]. At the same time, evidence indicates that nursing students’ readiness for AI‐integrated practice remains uneven. While students often report generally positive attitudes toward AI and its potential usefulness [10, 11], several studies have identified limited knowledge of AI concepts, weak understanding of practical applications, and insufficient awareness of the ethical issues associated with AI use in nursing and healthcare [12, 13]. This pattern is important because positive attitudes alone may not be enough to support appropriate and responsible AI utilization. Students may be willing to use AI, yet still lack the ethical understanding needed to evaluate its risks, limitations, and professional implications.

This gap points to the need for a more explanatory approach to understanding AI utilization among nursing students. Rather than focusing only on whether students hold favorable attitudes toward AI, it is important to examine what may help translate those attitudes into actual use. Guided by Social Cognitive Theory (SCT) [14], this study considers AI utilization as a behavioral outcome shaped by the interaction of personal cognition and self‐regulatory processes. Within this framework, AI ethics awareness can be understood as an important cognitive and moral mechanism that informs how students judge, adopt, and engage with AI‐related tools and practices. Students with greater ethical awareness may be better able to recognize issues such as bias, privacy, accountability, and patient safety and may, therefore, be more likely to use AI in ways that are thoughtful, professionally aligned, and responsible. By contrast, favorable attitudes in the absence of ethical awareness may not lead to appropriate utilization.

Examining the mediating role of AI ethics awareness is particularly relevant to nursing management. As healthcare organizations and educational institutions prepare for AI‐enabled practice, nurse leaders, academic administrators, and curriculum planners are increasingly responsible for ensuring that the future workforce is both digitally prepared and ethically grounded. Understanding whether ethics awareness helps explain the relationship between AI attitudes and AI utilization may, therefore, offer practical insight for curriculum design, leadership development, and governance strategies intended to support safe and responsible AI adoption in nursing education and practice.

Accordingly, this study aimed to examine the relationships among AI attitudes, AI ethics awareness, and AI utilization among nursing students from multiple study sites in Saudi Arabia, with particular attention to the mediating role of AI ethics awareness. While several of these associations have been examined individually in previous research, this study seeks to extend the literature by testing them within an integrated, theoretically informed model and by exploring the mediating role of AI ethics awareness in the link between AI attitudes and AI utilization. In doing so, it provides context‐specific evidence from Saudi Arabia and highlights implications for nursing education and management in preparing an ethically grounded and AI‐ready future workforce.

## 2. Background

AI technology attitudes refer to individuals’ willingness to accept, adopt, and utilize AI‐driven technologies and their perceptions of how these technologies influence society [15]. Within SCT, attitudes are considered key personal cognitive determinants that shape behavioral intentions but require mediating cognitive and moral processes to result in actual behavior. A systematic review of 22 studies involving nursing, medical, and dental students reported average AI knowledge alongside generally positive AI attitudes [16], indicating openness toward AI while highlighting the need for stronger curricular integration in healthcare education programs [6]. Similarly, a scoping review revealed that health science students were interested in and willing to engage with AI technologies, with AI attitudes directly associated with AI utilization and intention to use [17]. Nonetheless, perceived threats to job security and insufficient realism of AI software contributed to negative attitudes among some students [17].

Evidence from nursing education across different countries further illustrates variability in AI attitudes and utilization. Turkish nursing students with positive AI attitudes demonstrated greater readiness to use AI technologies in nursing practice [18]. Among Korean nursing students, positive AI attitudes combined with facilitating conditions such as self‐efficacy, performance expectancy, and effort expectancy significantly predicted intention to use AI [19]. Personality traits have also been associated with AI attitudes, with openness predicting more positive attitudes and neuroticism, agreeableness, and conscientiousness predicting more negative attitudes toward AI [11]. Demographic factors likewise influence AI attitudes. For example, first‐year male Croatian nursing students reported more positive attitudes toward AI use in nursing education [10], while among senior Saudi nursing students, positive AI attitudes were closely linked to higher levels of digital literacy [20]. Collectively, these findings indicate that AI attitudes play a pivotal role in shaping nursing students’ intentions toward AI use but do not consistently guarantee actual utilization.

AI ethics awareness refers to students’ awareness and appraisal of the ethical implications of AI use, including issues such as bias, privacy, accountability, transparency, and patient safety [19, 21]. In this study, it is conceptualized as a cognitive‐moral appraisal construct rather than as a behavior, a general moral trait, or a measure of technical competence. This distinction is important because the construct is intended to capture whether students recognize and consider the ethical dimensions of AI use, not whether they have already demonstrated ethical behavior in practice. Within the SCT framework, ethical awareness functions as a form of moral cognition and self‐regulation that may shape whether attitudes are translated into behavior. Empirical studies outside nursing have shown that AI ethics awareness is associated with ethical reasoning and higher‐order thinking, as demonstrated among Chinese primary school students [22] and with AI awareness and attitudes among Taiwanese nonengineering students [23]. Within nursing education, AI ethics has also been associated with more positive AI attitudes and greater self‐efficacy, while reducing anxiety related to AI use [19]. Taken together, these findings suggest that AI ethics awareness may serve as an intervening mechanism through which students evaluate the appropriateness of AI use and engage with AI in a more responsible and professionally aligned manner.

Recent evidence from diverse cultural and clinical contexts highlights the relevance of AI in nursing education and practice, emphasizing the importance of ethical awareness in its adoption. Among Egyptian nursing students, positive attitudes toward AI stem from perceptions of its usefulness and ease of use; gender, prior AI training, and academic level are key predictors of acceptance [24]. In Saudi Arabia, research finds that acceptance and perceived usability of AI are positively associated with students’ motivation for critical thinking, underscoring the value of early, structured AI training in fostering digital competence, and cognitive skill development [25].

Beyond educational settings, evidence from clinical contexts further illustrates the ethical and managerial dimensions of AI integration. A narrative review of mental health nursing highlights that AI may enhance early detection, risk assessment, treatment adherence, and remote patient monitoring; however, it also stresses the need to balance technological efficiency with human‐centered care, data privacy, and ethical responsibility [26]. In critical care environments, ethical concerns—such as overreliance, workflow adaptation, transparency, and accountability—are identified as central factors for building trust and ensuring responsible AI‐supported decision‐making [27]. Empirical findings among Egyptian nurses reveal that ethical awareness significantly moderates the relationship between nurses’ attitudes toward AI and their innovative work behaviors. Thus, ethics serves as the key mechanism linking positive AI attitudes to responsible and meaningful technology use [28]. Collectively, these culturally diverse findings support the need to examine AI ethical awareness as a mediating factor between AI attitudes and AI utilization among nursing students preparing for technology‐integrated healthcare systems.

Despite the growing body of empirical research on AI attitudes and utilization, studies examining AI ethics awareness as a mediating mechanism between AI attitudes and AI utilization remain scarce, particularly among nursing students and within Middle Eastern contexts. Understanding this mediating relationship is essential for informing nursing education strategies and leadership decisions aimed at preparing future nurses for ethically grounded AI adoption. To the best of our knowledge, this study is the first to investigate the mediating role of AI ethics awareness between AI technology attitudes and AI utilization among nursing students, offering a theoretically grounded and empirically informed contribution to nursing education and management literature.

### 2.1. Theoretical Framework

This study is grounded in Bandura’s SCT [14], which explains human behavior as the result of reciprocal interactions among personal factors, cognitive processes, and behavioral enactment. A central premise of SCT is that behavior is not determined by attitudes alone; rather, attitudes influence action through intervening cognitive and self‐regulatory processes that shape how individuals interpret situations, judge consequences, and regulate conduct [14, 29]. This perspective provides a useful framework for examining how nursing students’ attitudes toward AI may be associated with their actual AI utilization.

In the context of AI in nursing education, AI attitudes represent cognitive evaluations and beliefs regarding AI’s usefulness, risks, and relevance to clinical and managerial practice [18, 19, 30]. These attitudes may create general openness or resistance toward AI, but they do not by themselves explain whether students will use AI in a responsible and professionally appropriate manner. SCT emphasizes the role of internal appraisal and self‐regulatory judgment in translating beliefs into action [14, 29]. In this study, AI ethical awareness is conceptualized as this intervening cognitive‐moral process.

More specifically, AI ethical awareness refers to students’ awareness and appraisal of the ethical implications of AI use, including issues such as bias, privacy, accountability, transparency, and patient safety [21]. It is not treated here as a behavior, nor as a general moral trait, but as a cognitive‐moral evaluative construct that helps students judge whether, when, and how AI should be used in ways consistent with professional nursing values and standards [6]. Conceptualizing ethical awareness in this way reduces ambiguity and clarifies its role within the model.

Based on SCT, the proposed mediation model assumes that favorable AI attitudes may increase students’ willingness to engage with AI but that this willingness is more likely to translate into actual utilization when students are also able to recognize and evaluate the ethical dimensions of AI use. In other words, attitudes may foster receptiveness to AI, whereas ethical awareness helps regulate and channel that receptiveness into responsible behavioral enactment. This conceptualization is consistent with SCT’s assertion that behavior is shaped not only by attitudes but also by internalized cognitive and self‐regulatory mechanisms [14, 29].

Accordingly, AI ethical awareness is positioned in this study as a mediating mechanism between AI attitudes and AI utilization. This mediation is theoretically plausible because ethical awareness provides the evaluative link between students’ general perceptions of AI and their decision to use AI tools in educational or practice‐related contexts. Students who view AI positively but lack ethical awareness may remain hesitant, uncritical, or inconsistent in their use. By contrast, students with favorable attitudes and stronger ethical awareness may be better prepared to engage in AI utilization in a deliberate, accountable, and professionally aligned manner [19, 27].

**FIGURE 1:**
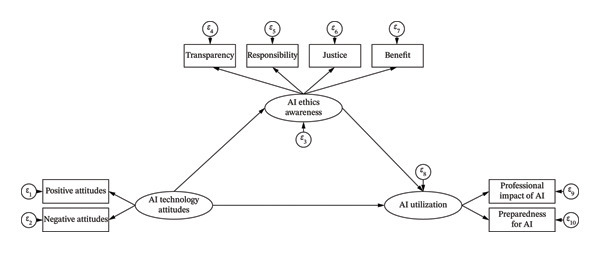
Theoretical and hypothesized model.

Thus, SCT supports a model in which AI attitudes function as a personal cognitive factor, AI ethical awareness functions as an intervening cognitive‐moral appraisal process, and AI utilization represents the behavioral outcome. Framing the model in this way strengthens its conceptual coherence and clarifies why ethical awareness is examined as a mediator rather than as a parallel predictor or outcome variable (Figure 1). Thus, the proposed model is theory informed; however, given the cross‐sectional nature of the data, the hypothesized pathways should be interpreted as associative rather than causal.

### 2.2. Study Hypotheses

After an extensive literature review, this investigation is anchored on the following hypotheses (Figure 1): (1) attitudes toward AI technology positively influence AI ethics awareness, (2) attitudes toward AI technology positively influence AI utilization, (3) AI ethics awareness positively influences AI utilization, and (4) AI ethics awareness mediates the association between attitudes toward AI technology and AI utilization.

## 3. Methods

### 3.1. Design

This investigation employed a cross‐sectional and correlational design while adhering to the STROBE guidelines in reporting the study’s results. Structural equation modeling (SEM) was used to display the interrelationships of study variables.

### 3.2. Setting, Sampling, and Participants

The study settings were four nursing colleges spread across three regions of Saudi Arabia. Study Setting 1 is the only private nursing college in the northern province. Study Setting 2 has a nursing program with its own medical hospital as its internship training center located in the western region. Also, in the western region, Study Setting 3 is a part of state university. Study Setting 4 is a constituent of a state university in the central region.

The sampling frame consisted of undergraduate nursing students enrolled in the participating colleges during the data collection period. Consecutive sampling was used, whereby all eligible students at each site were invited to participate sequentially during the study period [31]. The eligibility criteria were (a) officially matriculated student nurses in the study settings, (b) finished at least one semester, and (c) consented to participate. Recruitment was facilitated through institutional coordination, and students who met the inclusion criteria were invited to complete the online survey. This approach aimed to maximize participation across sites while maintaining feasibility within academic settings. Although consecutive sampling does not ensure random selection, its use across multiple institutions helped broaden representation and reduce the likelihood of single‐site sampling bias [31].

The sample size calculation was in accordance to the assumption of Hair and Alamer [32] for an SEM analysis that for every item in each scale is multiplied by 10 (i.e., 20 + 12 + 10 = 42 ∗ 10 = 420). A total of 1000 survey forms were distributed to potential participants, and 827 data entries were received for analysis (response rate: 82.70%). However, 62 entries were invalid as participants did not consent to participate. Hence, these were omitted from the analyses. A total of 765 valid data entries were included in the final analyses, with no missing data. Hence, the final sample size satisfied the requirements for a SEM analysis.

### 3.3. Ethical Considerations

Ethical approval for this study was obtained from the Ethical Research Committee of the North Private College of Nursing (serial no.: NCN‐22102024‐15, approval date: 24 October 2024) prior to data collection. All study procedures were conducted in accordance with recognized ethical standards for research involving human participants.

Participation in the study was entirely voluntary. Data were collected using an online survey platform that included a comprehensive electronic informed consent form presented on the first page of the survey. The consent form clearly explained the study’s purpose, procedures, expected duration, potential benefits and risks, and participants’ rights, including the right to decline participation or withdraw at any time without penalty. Participants were informed that no incentives or academic consequences were associated with participation. Informed consent was obtained through an implied consent process, whereby participants indicated their agreement by clicking the “I consent to participate” option and submitting the completed survey [31]. Participants were informed that they could decline to answer any survey item and could withdraw from the study at any time without penalty or negative consequences.

To protect participants’ privacy and confidentiality, no personally identifiable information was collected. Data were anonymized at the point of collection and stored securely on a password‐protected laptop accessible only to the researchers. All electronic data were handled in compliance with institutional data protection policies and were used solely for research purposes. The researchers ensured adherence to core ethical principles, including respect for autonomy (voluntary participation and informed consent), confidentiality and anonymity (secure data handling and exclusion of personal identifiers), respect for persons (acknowledging participants’ right to refuse or withdraw), and nonmaleficence (minimizing potential psychological or social risks). Participants were informed that the study posed no anticipated physical harm and minimal risk. Furthermore, the study was conducted in accordance with the 2024 Declaration of Helsinki [33], ensuring ethical integrity throughout the research process.

### 3.4. Measures

The survey questionnaire was composed of four sections. The first section obtained the participants′ demographic data (e.g., age, sex, year level, and grade point average [GPA]). Section 2 included the 20‐item General Attitudes Toward Artificial Intelligence Scale (GATAIS) [15]. Section 3 comprised the 12‐item AI Ethics Awareness Scale (AIEAS) [23]. Section 4 contained the 10‐item Perceptions Regarding AI Utilization Scale (PRAIU) [34].

Nursing students’ attitudes toward AI were assessed using the 20‐item GATAIS [15]. This scale has two subscales: positive (12 items) and negative (8 items) attitudes. The scale is rated on a 5‐point Likert scale (1 = *strongly disagree* to 5 = *strongly agree*). The items about negative attitudes are initially reverse coded; hence, the higher scores depict positive attitudes toward AI. The scale’s former alphas were 0.88 and 0.83 for positive and negative attitudes, respectively [15]. This instrument was recently used among Korean student nurses, where the scale reported alphas of 0.85 (positive attitudes) and 0.76 (negative attitudes) [19].

The 12‐item AIEAS was utilized to determine nursing students’ AI ethics awareness [23]. This scale was a concise version of the Jobin et al.’s [35] AI ethics scale. This scale has 4 dimensions (transparency, responsibility, justice, and benefit), each containing 3 items. Each item is scored using the 5‐point Likert scale ranging from 1 (*strongly disagree*) to 5 (*strongly agree*). The total score ranges from 12 to 60. A higher score indicates excellent AI ethics awareness. The overall alphas of this scale were 0.91, indicating that the scale has excellent reliability [23].

The 10‐item PRAIU was used to ascertain nursing students’ perceptions of AI utilization [34]. The primary objective of this scale is to ascertain participants’ self‐perceived integration and applicability of AI in healthcare [34]. This scale contains two factors: Professional impact of AI (items 1 to 5 and 7) and Preparedness for AI (Items 6 and 8–10). A 5‐point Likert scale (1 = *strongly disagree* to 5 = *strongly agree*) was used to rate each item. The score is computed by estimating the average or mean scores. The overall mean scores are divided into three types: 1.00–2.33 (low perceptions), reflecting that nursing students show or apprehension or uncertainties about AI’s role in nursing and may be doubtful to adopt AI technologies; 2.34–3.66 (moderate perceptions), denoting that nursing students are open to the AI’s use in nursing but have concerns or need additional knowledge in accepting its encompassing possibilities; and 3.67–5.00 (high perceptions), representing nursing students’ optimistic view of AI and believe in its benefits. The scale’s original alpha was 0.80, indicating good reliability [34].

### 3.5. Validity and Reliability

Three validated self‐report instruments were used to measure AI technology attitudes, AI ethics awareness, and perceived AI utilization. Permission to use and adapt the instruments was obtained from the original authors before data collection. The scales were translated from English into Arabic using a forward–backward translation process. Three bilingual university professors independently translated the instruments into Arabic, and three independent language experts, blinded to the original versions, backtranslated them into English. Any discrepancies were resolved by expert consensus to ensure conceptual equivalence.

Content validity was assessed by three experts using the Content Validity Index (CVI). After two rounds of review and revision, the CVI values were 0.92 for the GATAIS, 0.91 for the AIEAS, and 0.93 for the PRAIU, indicating acceptable content validity. The Arabic versions were then pilot tested with 25 nursing students to assess internal consistency before the main study. Cronbach’s alpha coefficients were 0.93 for GATAIS, 0.96 for AIEAS, and 0.94 for PRAIU, indicating good reliability.

### 3.6. Data Collection

Data collection was conducted over a 3‐month period (November 10, 2024–April 27, 2025). Following the acquisition of ethical clearance, formal authorization was secured from the relevant college authorities (e.g., the dean and administrator). The survey was administered through a Google online form, and invitations containing the survey link were distributed via the students’ institutional email accounts. To maximize response rates, weekly email reminders were disseminated and class leaders were requested to announce the study to their cohorts. No incentives were provided to participants. The research team emphasized that participation was entirely voluntary and that withdrawal from the study at any stage would have no adverse consequences for academic standing.

### 3.7. Data Analysis

All statistical analyses were performed using STATA MP (Parallel Edition, Version 18; StataCorp, College Station, TX). A *p* value of < 0.05 was considered statistically significant. Descriptive statistics (mean, standard deviation, frequency, and percentage) were used to summarize the study variables. Data normality was assessed using the Shapiro–Wilk and Doornik–Hansen tests. Correlation analyses of associations among AI technology attitudes, ethics awareness, and utilization were initially conducted using Pearson’s r [36]. Subsequently, covariance‐based structural equation modeling (CB‐SEM) with maximum likelihood estimation with Satorra–Bentler adjustment, to compensate for nonnormality, was applied to test the theoretical model of interrelationships among these constructs. Model fit of the emerging and hypothesized model were evaluated using the following indices and threshold values: *χ* ^2^/df ≤ 3.00, root mean square error of approximation (RMSEA) ≤ 0.08 [37], comparative‐fit index (CFI) and Tucker–Lewis index (TLI) ≥ 0.90, and standardized root mean squared residual (SRMR) ≤ 0.10 [38]. Finally, mediation analysis was performed through path analysis to examine the indirect effect of AI ethics awareness. The significance and stability of path coefficients were appraised using bootstrapping procedures (5000 resamples and bias‐corrected confidence intervals at 95% level). Sensitivity analyses according to sex and year level were also conducted to determine the invariance across groups. Because this study used a cross‐sectional design, the mediation analysis was conducted to examine indirect statistical relationships among AI attitudes, AI ethics awareness, and AI utilization rather than to establish temporal or causal pathways.

## 4. Results

### 4.1. Participants’ Demographic Characteristics

The demographic characteristics of the participants are presented in Table 1. It can be noted that the mean age of the participants was 23.98 years old (SD = 4.40), and majority were females (78.82%), were second year students (33.86%), and had a mean academic performance, measured using their GPA, of 4.16 (SD = 0.65).

**TABLE 1:** Demographic characteristics of the participants (*n* = 765).

Characteristics	Frequency (*f*)	Percentage (%)	Mean (SD)
Age (Years)			23.98 (4.40)
Sex			
Male	162	21.18	
Female	603	78.82	
Study Settings			
Study Setting 1	455	59.48	
Study Setting 2	72	9.41	
Study Setting 3	113	14.77	
Study Setting 4	125	16.34	
Year level			
First year	209	27.32	
Second year	259	33.86	
Third year	190	24.84	
Fourth year	107	13.98	
Academic performance (grade point average)			4.16 (0.65)

### 4.2. Descriptive Statistics of the Study Variables

Table 2 shows the descriptive statistics of AI technology attitudes, AI ethics awareness, and AI utilization and their underlying dimensions. The overall AI technology attitudes, ethics awareness, and utilization can all be interpreted as moderately high. For the dimensions of AI technology attitudes, there was a high positive and low negative attitudes toward AI technology. In a different light, all four dimensions of AI ethics awareness and AI utilization can be interpreted as high levels.

**TABLE 2:** Descriptive statistics of AI technology attitudes, AI ethics awareness, and AI utilization among the participants (*n* = 765).

Variables	Mean ($\overset{¯}{x}$)	SD	Score range
Artificial intelligence (AI) technology attitudes	**3.20**	**0.51**	**1.00**–**5.00**
Positive attitudes	3.88	0.77	1.00–5.00
Negative attitudes	2.52	0.96	1.00–5.00
Artificial intelligence (AI) ethics awareness	**3.86**	**0.78**	**1.00**–**5.00**
Transparency	3.85	0.89	1.00–.00
Responsibility	3.85	0.84	1.00–5.00
Justice	3.90	0.82	1.00–5.00
Benefits	3.85	0.85	1.00–5.00
Artificial intelligence (AI) utilization	**3.72**	**0.77**	**1.00**–**5.00**
Professional impact of artificial intelligence (AI)	3.72	0.79	1.00–5.00
Preparedness for artificial intelligence (AI)	3.73	0.84	1.00–5.00

*Note:* Bold values signify overall mean, standard deviation (SD), and score range.

### 4.3. Associations Among Study Variables

Table 3 illustrates the correlation and linear associations of the dimensions of AI technology attitudes, AI ethics awareness, and AI utilization. The dimension of positive attitudes under AI technology attitudes had moderately strong, positive associations with the dimensions of AI ethics awareness and AI utilization. In contrast, the negative attitudes domain had moderately strong, negative associations. Analyses also indicated that the four domains of AI ethics awareness has strong, positive associations with the two dimensions of AI utilization.

**TABLE 3:** Correlation coefficients of the associations of AI technology attitudes, AI ethics awareness, and AI utilization among the participants (*n* = 765).

Variables	1	2	3	4	5	6	7	8
1. Positive attitudes (AI technology attitudes)	—	—	—	—	—	—	—	—
2. Negative attitudes (AI technology attitudes)	−0.32^∗^ (0.001)	—	—	—	—	—	—	—
3. Transparency (AI ethics awareness)	0.45^∗^ (0.001)	−0.32^∗^ (0.001)	—	—	—	—	—	—
4. Responsibility (AI ethics awareness)	0.50^∗^ (0.001)	−0.37^∗^ (0.001)	0.75^∗^ (0.001)	—	—	—	—	—
5. Justice (AI ethics awareness)	0.50^∗^ (0.001)	−0.38^∗^ (0.001)	0.76^∗^ (0.001)	0.86^∗^ (0.001)	—	—	—	—
6. Benefits (AI ethics awareness)	0.54^∗^ (0.001)	−0.37^∗^ (0.001)	0.68^∗^ (0.001)	0.81^∗^ (0.001)	0.84^∗^ (0.001)	—	—	—
7. Professional impact of AI (AI utilization)	0.56^∗^ (0.001)	−0.41^∗^ (0.001)	0.66^∗^ (0.001)	0.73^∗^ (0.001)	0.75^∗^ (0.001)	0.76^∗^ (0.001)	—	—
8. Preparedness for AI (AI utilization)	0.50^∗^ (0.001)	−0.38^∗^ (0.001)	0.66^∗^ (0.001)	0.66^∗^ (0.001)	0.69^∗^ (0.001)	0.73^∗^ (0.001)	0.80^∗^ (0.001)	—

*Note:* Values are presented as *r*‐value (*p* value).

^∗^Significant at 0.05.

### 4.4. Validity and Reliability of Measurement Models

The measurement models showed acceptable reliability and validity. For AI technology attitudes (i.e., GATAIS), factor loadings ranged from 0.42 to 0.86, Cronbach’s alpha was 0.927, composite reliability (CR) was 0.958, and average variance extracted (AVE) was 0.540. For AI ethics awareness (i.e., AIEAS), factor loadings ranged from 0.76 to 0.94, Cronbach’s alpha was 0.959, CR was 0.973, and AVE was 0.749. For AI utilization (i.e., PRAIU), factor loadings ranged from 0.71 to 0.86, Cronbach’s alpha was 0.942, CR was 0.940, and AVE was 0.613. Discriminant validity was supported by heterotrait–monotrait (HTMT) values below 0.90, which ranged from 0.317 to 0.88. Overall, these findings indicate acceptable internal consistency, convergent validity, and discriminant validity for all three constructs.

### 4.5. Assessment of Common Method Bias (CMB)

Because the data were collected using self‐report questionnaires, CMB was assessed. Harman’s single‐factor test showed poor fit for a one‐factor model, suggesting that CMB was unlikely to account for most of the variance. However, the latent common method factor approach indicated a significant method effect (*p* = 0.001). Therefore, a common method factor was included in the subsequent analyses to control for potential bias.

### 4.6. Hypothesized Model of the Study Variables’ Associations

Given the nature of the data collection measures, statistical checks for CMB were conducted. Albeit the Harman test suggested no CMB for the three variables, the common latent method factor analyses suggested CMB for AI technology attitudes, AI ethics awareness, and AI utilization (*p* < 0.05). Hence, the common method variables were included in the analyses to control for CMB. Figure 1 illustrates the path diagram of the hypothesized model for the interrelationships of AI technology attitudes, AI ethics awareness, and AI utilization. Analysis of the hypothesized model indicated that it had acceptable model fit indices (*X* ^2^/df = 2.59, RMSEA = 0.059, CFI = 0.992, TLI = 0.985, and SRMR = 0.014) since the parameters were within the acceptable thresholds (Table 4). Nevertheless, modification indices recommended a covariance term between the domains of transparency and benefits (MI = 27.46 and EPC = 0.35) and between responsibility and justice (MI = 21.68 and EPC = 0.44), which are also supported theoretical underpinnings. These preliminary results were used to respecify the model.

**TABLE 4:** Model fit parameters with Satorra–Bentler adjustment of the hypothesized and emerging models (*n* = 765).

Model	CMIN			RMSEA 90% CI			CFI	TLI	SRMR
	*χ* ^2^	df	*χ* ^2^/df (*p* value)	RMSEA (*p* value)	Lower bound	Upper bound			
Acceptable threshold	—	—	≤ 3.00 (> 0.05)	≤ 0.08 (> 0.05)	—	—	≥ 0.90	≥ 0.90	≤ 0.10
Hypothesized model	30.00	17	1.76 (0.026)	0.041 (0.049)	0.050	0.091	0.993	0.988	0.018
Emerging model	11.50	15	0.77 (0.716)	0.001 (0.886)	0.001	0.056	1.000	1.004	0.010

*Note:* *χ* ^2^ = chi‐square.

Abbreviations: CFI = Comparative Fit Index, df = degrees of freedom, RMSEA = root mean square error of approximation, SRMR = standardized root mean squared residual, TLI = Tucker–Lewis Index.

### 4.7. Emerging Model of the Study Variables

**FIGURE 2:**
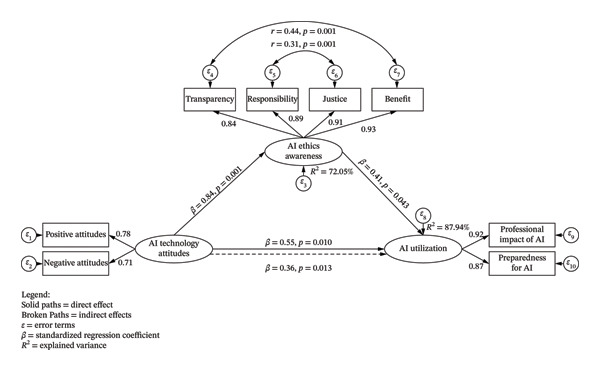
Emerging model.

The emerging model of associations of AI technology attitudes, AI ethics awareness, and AI utilization is presented in Figure 2. After one iteration of model refitting to include the recommended covariance terms, the emerging model yielded better model fit parameters as presented in Table 4. Satorra–Bentler adjustment (*X* ^2^ = 11.50 and *p* = 0.716) was not statistically significant, indicating good model fit despite nonnormality of data.

It can be noted in the figure that the dimensions of positive and negative attitudes of AI technology attitude had factor loadings of 0.78 and 0.71, respectively, and had Cronbach’s alpha values of 0.928 and 0.936, respectively. Results also showed that the CR was 0.850, while the AVE was 0.556. For the four dimensions of AI ethics awareness, the factor loadings ranged from 0.84 to 0.93 and the reliability coefficients were 0.886 for transparency, 0.904 for responsibility, 0.893 for justice, and 0.911 for benefit. The computed CR was 0.955 and the AVE was 0.798. The first dimension of AI utilization, professional impact of AI, had a factor loading of 0.92 and a Cronbach’s alpha of 0.906, while the second factor (preparedness for AI) had a loading of 0.87 and a reliability coefficient of 0.887. The CR was 0.954, while the AVE was 0.802. The HTMT ratio criteria between AI technology attitudes and AI ethics awareness and AI utilization were 0.861 and 0.821, respectively, while it was 0.895 between AI ethics awareness and AI utilization. From these results, it can be inferred that AI technology attitude, AI ethics awareness, and AI utilization had acceptable internal consistency and convergent validity since factor loadings, Cronbach’s alpha, and CR were ≥ 0.70 and the AVE estimates were > 0.50. Likewise, discriminant validity was ascertained cognizant that all three HMTM criteria were < 0.90.

The model demonstrates that AI technology attitudes positively and directly associated with AI ethics awareness (*β* = 0.84, *p* = 0.001) and AI utilization (*β* = 0.55, *p* = 0.010). AI ethics awareness, on the other hand, had a direct and positive association with AI utilization (*β* = 0.41, *p* = 0.043). The mediation analysis indicated a significant indirect association between AI attitudes and AI utilization (*β* = 0.36, *p* = 0.013) through AI ethics awareness (Table 5). These findings reflect statistical relationships among the variables and should not be interpreted as evidence of causation. AI technology attitudes measured 72.05% of the explained variance of AI ethics awareness, while both AI ethics awareness and AI technology attitudes measured 87.94% of the variance of AI utilization. The magnitude of the standardized beta coefficients indicates moderate to strong associations among AI attitudes, AI ethical awareness, and AI utilization, suggesting that these constructs are meaningfully related rather than trivially associated.

**TABLE 5:** Path analyses of the total, direct, and indirect effects among AI technology attitudes, AI ethics awareness, and AI utilization among the participants (*n* = 765).

Paths	*β* coefficient	Bias‐corrected 95% CI	*p* value (two‐tailed)
Direct effects			
AI Technology Attitudes ⟶ AI Ethics Awareness	0.84^∗^	0.76 to 0.94	0.001
AI Technology Attitudes ⟶ AI Utilization	0.41^∗^	0.01 to 0.84	0.043
AI Ethics Awareness ⟶ AI Utilization	0.55^∗^	0.13 to 0.97	0.010
Indirect effects			
AI Technology Attitudes ⟶ AI Ethics Awareness ⟶ AI Utilization	0.36^∗^	0.05 to 0.68	0.013
Total effects			
AI Technology Attitudes ⟶ AI Utilization	0.91^∗^	0.55 to 1.27	0.001

*Note:* Values are presented as standardized regression or beta coefficient (*p* value).

^∗^Significant at 0.05 level.

### 4.8. Sensitivity Analyses

Sensitivity analyses according to the sex (*χ* ^2^ = 1.34 and *p* = 0.720) and year level (*χ* ^2^ = 5.72 and *p* = 0.768) suggested that the regression coefficients were invariance and not significantly different across sex and year levels. These results suggest that the associations among AI technology attitudes, AI ethics awareness, and AI utilization were not significantly affected by the sex and year level of participants.

## 5. Discussion

This study examined the associations among nursing students’ AI attitudes, AI ethics awareness, and AI utilization, with particular attention to the statistical mediating role of AI ethics awareness. Four main findings were observed. First, positive attitudes toward AI were significantly associated with both AI ethics awareness and AI utilization. Second, AI ethics awareness was significantly associated with AI utilization. Third, AI ethics awareness showed a significant mediating relationship in the association between AI attitudes and AI utilization. Lastly, AI attitudes explained 72.05% of the variance in AI ethics awareness, whereas AI attitudes and AI ethics awareness together explained 87.94% of the variance in AI utilization.

Although several of the observed associations are consistent with previous literature, the contribution of this study lies in examining how these variables operate together within a theoretically informed model. Rather than simply confirming that attitudes, ethics awareness, and utilization are related, the findings suggest that AI ethics awareness may help explain how favorable attitudes toward AI are associated with students’ reported AI utilization. This provides a more nuanced account of AI readiness among nursing students and identifies ethical awareness as a potentially actionable focus for nursing management and educational intervention.

The findings revealed that AI technology’s positive attitudes directly linked to AI ethics awareness and AI utilization (validating Hypotheses 1 and 2). Hence, providing empirical evidence that positive AI attitudes could develop ethical awareness and facilitate AI use. These results imply two key points. First, positive attitudes toward AI technology enhance nursing students’ AI ethics awareness. Second, students are more inclined to use AI when they have positive attitudes toward AI technology. These findings support previous studies [18, 39, 40]. Korean [39] and Jordanian [40] nursing students showed that favorable AI attitudes improve the understanding of AI ethics. Moreover, nursing students from Turkey demonstrated readiness to use AI‐integrated technologies in nursing due to their positive attitudes toward AI [18].

Positive AI attitudes also foster self‐efficacious behaviors and improved academic performance [19]. Hence, this suggests that to inspire attitudes toward AI technology, nursing students must understand the use and adaptation of technologies influenced by AI; therefore, emphasizing the roles of education and experience in shaping students’ understanding and eagerness to use AI [15, 41]. These attitudes are critical for cultivating ethical practices when using AI technologies and for making decisions about their use.

Nursing students are better equipped to provide safe, high‐quality patient care when they are competent in operating AI‐integrated technologies in any healthcare setting and are mindful of the ethical principles in applying AI. Nurse academics should take a proactive approach to shaping nursing students’ attitudes toward AI. It is important to cultivate a greater appreciation for AI, which will enhance students’ engagement and willingness to integrate AI in their clinical placements. Furthermore, addressing any negative attitudes arising from misconceptions or fear of technology is equally crucial.

The results show a direct association between student nurses’ awareness of AI ethics and their intent to use AI. This confirms Hypothesis 3. Ethical awareness improves the use of AI in academic and clinical settings. Prior studies support this finding [22, 23, 40]. AI ethics awareness enhances students’ critical thinking and helps them judge when AI technologies are necessary [22, 23]. This awareness is vital for responsible AI use. Nursing relies on ethical practice for patient care [42, 43], which AI tools cannot replicate. While AI supports nursing tasks, it lacks the ethical understanding and compassion needed for patient care. Therefore, nursing students must develop strong knowledge of AI ethics to maintain high ethical and professional standards. Future nurses must respond appropriately when AI breaches ethical principles, such as data leaks, flawed treatment algorithms, or equipment mismatches. These risks highlight the need for nursing education institutions to offer continuous programs. Educators may need to train students on AI ethics and responsible AI use in healthcare settings.

Focusing on the emerging model, AI ethics awareness mediated the association between AI technology attitudes and AI utilization. This affirms Hypothesis 4. This suggests that favorable attitudes toward AI were associated with greater AI utilization partly through higher AI ethics awareness. Previous studies only showed links between AI ethics awareness and attitudes or utilization [28, 30, 40, 41]. To our knowledge, this study is the first to report the mediating role of AI ethics awareness. From a nursing management perspective, this finding is particularly important because it suggests that positive attitudes alone may not be sufficient for responsible AI readiness. Students may be more likely to engage appropriately with AI when they are also able to recognize ethical issues such as accountability, transparency, bias, and patient safety. This means that nurse leaders, academic administrators, and faculty managers should consider AI ethics awareness as an operational element of workforce preparation, curriculum governance, and responsible technology integration. The results presented evidence that AI ethics awareness fosters mechanisms that support nursing students’ positive attitudes and appropriate AI use. The emerging model also highlights a strong direct relationship between nursing students’ attitudes toward AI technology and their awareness of AI ethics. Although the findings are consistent with the proposed mediation model, the cross‐sectional design does not permit conclusions about directionality or causality. Accordingly, AI ethical awareness should be interpreted as a statistical mediator in this study rather than a confirmed causal mechanism.

The findings show that nursing students’ attitudes explain 72.05% of their AI ethics awareness. This indicates a strong statistical association between students’ perceptions of AI and their ethical awareness of its use. From a nursing management perspective, this finding suggests that educational and leadership strategies aimed at AI readiness should extend beyond technical training alone. In turn, this awareness influences willingness to engage with AI technologies in hospital and clinical placements. For nursing educational institutions and managers, this highlights the need to embed AI ethics into curriculum planning, faculty development, and institutional guidance so that students are prepared to use AI in ways that are not only effective but also professionally accountable and safe. These findings are vital for nursing educational institutions. They encourage a focus on fostering both favorable attitudes and strong ethical understanding. By doing this, educators can better prepare students to competently and ethically integrate AI into their healthcare practices.

AI ethics includes fundamental principles that guide AI systems when processing external data [6]. These principles—accountability, autonomy, justice, and societal impact—help AI learn and self‐adjust to meet objectives responsibly [44]. In healthcare, integrating AI into nursing education requires a robust ethical framework. This framework should guide educators and empower students to handle the complexities of AI use in clinical settings [9]. AI ethics can also enhance academic integrity and protect data privacy and security [45]. Therefore, nursing institutions and educators should incorporate AI ethics into their curricula. This can be achieved by developing relevant policies and training educators to ensure effective implementation. These measures will foster students’ positive attitudes toward AI and encourage responsible use of technology. Prioritizing ethics will help nursing programs improve education and prepare professionals to manage AI challenges in healthcare.

Beyond statistical significance, the magnitude of the observed effects provides insight into the practical relevance of the findings. The standardized beta coefficients indicate moderate to strong relationships among AI attitudes, AI ethical awareness, and AI utilization. This suggests that these variables are associated not only at a statistical level but also at a level that may be meaningful in educational and managerial practice. For nursing management and education, these effect sizes imply that targeting ethical awareness and attitudes may support tangible improvements in how future nurses engage with AI technologies. In practical terms, this highlights the value of interventions such as curriculum revision, ethics‐focused AI education, and faculty support strategies aimed at promoting responsible AI use.

The high proportion of variance explained in AI utilization (*R* ^2^ = 87.94) indicates that 87.94% of the variability in nursing students’ AI utilization is attributable to the combined effects of AI technology attitudes and AI ethics awareness. From a practical perspective, this finding suggests that attitudinal and ethical factors are central, rather than peripheral, to understanding students’ AI engagement. While such a value may raise concerns about overfitting or construct redundancy, it can also reflect strong theoretical alignment among closely related psychosocial constructs. These constructs are measured within a coherent conceptual framework. In this study, AI attitudes, AI ethical awareness, and AI utilization were specified as interrelated but distinct constructs grounded in SCT [14, 29, 46]. For nursing management, this means that leadership efforts focused only on technical access or exposure may be insufficient. Programs may also need to strengthen students’ ethical appraisal and confidence in using AI responsibly. The use of validated instruments and acceptable reliability indices supports construct distinctiveness.

Comparable studies in nursing education and healthcare technology adoption have reported similarly strong explanatory power when attitudes, ethical considerations, and perceived usability are modeled together [11, 19, 24, 28, 39]. This is especially true in educational samples where contextual variability is limited and constructs are conceptually close. Thus, the observed *R* ^2^ value likely reflects the model’s explanatory power rather than statistical overfitting. At the same time, this result should be interpreted cautiously. The practical implication is not that AI utilization is fully determined by these variables but that attitudes and ethical awareness appear to be important leverage points for educational and managerial intervention within this study context. Even so, future research using longitudinal designs, alternative modeling approaches, or external validation samples is recommended. Such work would help determine whether these practically important associations remain stable across settings and over time.

### 5.1. Limitations and Recommendations

Several limitations were encountered in this study. First, the cross‐sectional design prevents causal inference, even though mediation analysis was performed. While the results support an indirect statistical relationship consistent with the proposed theoretical model, they do not establish that AI attitudes lead to greater AI ethics awareness or that ethics awareness, in turn, leads to increased AI utilization. Although this study employed a multisite design involving four nursing colleges across three regions, the use of consecutive sampling may limit the generalizability of the findings. While recruiting participants from multiple institutions helped reduce site‐specific bias, the nonrandom sampling approach may still have introduced selection bias, as participation depended on students’ availability and willingness to respond. In addition, variations in the enrollment size and educational contexts across sites may have influenced participation rates. Furthermore, the predominance of female participants, representing nearly 80% of the sample, may limit the broader applicability of the findings, particularly to male nursing students and to settings with more gender‐balanced populations.

The findings should be interpreted within the Saudi Arabian cultural and educational context, which may shape students’ perceptions of AI, ethical awareness, and patterns of AI utilization. Although the multisite design included nursing colleges across three regions, institutional and contextual differences may still have influenced the observed relationships. In particular, private and public institutions may differ in resource availability, governance structures, digital infrastructure, faculty readiness, and exposure to emerging technologies, all of which could affect students’ experiences with AI and AI ethics. In addition, curricular exposure to AI and AI ethics was not uniform across participating programs. Although multigroup SEM was conducted at a limited level, these institutional differences were not examined in sufficient analytical depth to determine whether the proposed relationships varied systematically across institutional types. Accordingly, caution is warranted when applying these findings to other educational systems, cultural contexts, or nursing programs with different levels of AI integration. Future research should replicate and extend this model across diverse cultural settings and should more thoroughly compare public and private institutions, including more robust multigroup analyses to assess structural differences across educational contexts.

The use of self‐report measures may be influenced by social desirability and response bias. The online survey design may also have introduced self‐selection bias, as participation was voluntary. In addition, variability in curricular exposure to AI and AI ethics across participating institutions may have affected students’ responses. These limitations undermine statistical power and the external and internal validity of results. This study included selected demographic variables, namely, age, year level, and GPA, which are relevant to academic development and cognitive engagement with AI technologies. However, other sociodemographic factors, such as socioeconomic status, prior exposure to AI training, and cultural background, were not examined. As a result, generalizability is only limited to the study participants, and results should be cautiously interpreted. In addition, albeit statistical measures were employed to control for CMB, residual bias is still likely.

Accordingly, future research may utilize experimental, prospective, or longitudinal studies with multigroup invariance testing and long‐term follow‐up to establish temporal ordering and causal pathways more rigorously, include diverse sample sizes from multiregions/countries while employing probability‐based sampling strategies, and report site‐specific enrollment and response rates to further strengthen generalizability. Future studies should consider incorporating a broader range of demographic and contextual variables to further elucidate factors influencing AI attitudes, ethical awareness, and utilization among nursing students. Other variables like AI literacy, self‐efficacy, or competency may be included to examine metrological interactions. A qualitative or mixed‐methods design may elucidate a deeper understanding of the study variables.

## 6. Conclusion

This study demonstrates significant positive associations among AI technology attitudes, AI ethics awareness, and AI utilization, with AI ethics awareness showing a statistical mediating role in the association between attitudes and utilization. Overall, the study contributes to the growing literature on AI in nursing education by moving beyond isolated bivariate relationships and testing an integrated model linking AI attitudes, AI ethics awareness, and AI utilization. In doing so, it identifies AI ethics awareness as an important explanatory and practical focus for nursing management strategies aimed at preparing a digitally competent and ethically grounded future workforce. From a nursing management perspective, these findings emphasize the importance of ethical awareness in guiding responsible AI adoption within educational and clinical settings. Nursing leaders and policymakers should prioritize integrating AI ethics into curricula and institutional governance to support the development of an ethically grounded, AI‐ready nursing workforce. Nurse managers and academic leaders can further leverage AI ethics to guide educators and clinical mentors in supporting students’ learning needs. Ongoing monitoring and evaluation of AI integration are essential to inform evidence‐based managerial decisions related to curriculum revision, resource allocation, and policy development.

### 6.1. Implications for Nursing Management

The findings of this study offer important implications for nursing management, particularly for nurse leaders, academic administrators, and policymakers responsible for curriculum governance and workforce development. Given AI ethical awareness’s mediating role between AI attitudes and AI utilization, nursing management must move beyond promoting positive attitudes toward AI and focus on embedding ethical competence as a core managerial priority.

At the institutional level, nursing education policymakers and academic leaders play a critical role in shaping curricula that integrate AI concepts with ethical frameworks. Nursing management should advocate for policies that formally incorporate AI ethics into nursing curricula, ensuring that students are not only exposed to AI technologies but are also equipped to evaluate their ethical implications in clinical and educational contexts. Such governance‐level decisions can support a structured and consistent approach to AI integration across nursing programs.

At the organizational and operational levels, nurse administrators in educational institutions and affiliated clinical training centers are responsible for creating learning environments that enable ethical engagement with AI. This includes allocating resources for AI‐supported teaching and learning materials, investing in simulation technologies, and ensuring access to AI‐trained faculty and clinical mentors. Nurse managers should also facilitate faculty development programs that enhance educators’ and clinical supervisors’ competence in AI concepts and ethical decision‐making, positioning them as role models for responsible AI use.

From a leadership and oversight perspective, nurse managers serve as critical links between policy and practice. Ongoing monitoring and evaluation of AI integration in nursing education and clinical training are essential to assess its impact on learning outcomes, ethical practice, and patient safety. Nurse leaders should establish mechanisms for continuous feedback and ethical review to ensure that AI technologies are used as supportive tools rather than substitutes for clinical judgment and professional accountability.

Finally, from a professional development standpoint, nursing management has a responsibility to foster a culture of ethical awareness among nursing students and early‐career nurses. Encouraging reflective practice, ethical deliberation, and responsible reliance on AI technologies can help prepare future nurses to engage critically with AI while maintaining professional autonomy. By prioritizing ethical awareness as a managerial and leadership concern, nursing management can play a pivotal role in shaping an AI‐ready nursing workforce aligned with professional values and patient‐centered care.

## Author Contributions

Conceptualization: Daniel Joseph E. Berdida, Noura Alhudaib, Rizal Angelo N. Grande, Salah A. Alsharafi, Cyrelle D. Agunod, Adelina M. Santos, Hanay Huwaydi Alanazi, Modi Al‐Moteri, Nada Alqarawi, and Ibrahim Alasqah. Methodology: Daniel Joseph E. Berdida, Noura Alhudaib, Rizal Angelo N. Grande, Salah A. Alsharafi, Cyrelle D. Agunod, Adelina M. Santos, Hanay Huwaydi Alanazi, Modi Al‐Moteri, Nada Alqarawi, and Ibrahim Alasqah. Software: Daniel Joseph E. Berdida. Validation: Daniel Joseph E. Berdida, Noura Alhudaib, Rizal Angelo N. Grande, Salah A. Alsharafi, Cyrelle D. Agunod, Adelina M. Santos, Hanay Huwaydi Alanazi, Modi Al‐Moteri, Nada Alqarawi, and Ibrahim Alasqah. Formal analysis: Daniel Joseph E. Berdida, Rizal Angelo N. Grande, Nada Alqarawi, and Ibrahim Alasqah. Investigation: Daniel Joseph E. Berdida, Noura Alhudaib, Salah A. Alsharafi, Cyrelle D. Agunod, Adelina M. Santos, and Hanay Huwaydi Alanazi. Data curation: Daniel Joseph E. Berdida, Salah A. Alsharafi, Cyrelle D. Agunod, Adelina M. Santos, and Hanay Huwaydi Alanazi. Writing–original draft: Daniel Joseph E. Berdida and Rizal Angelo N. Grande. Writing–review and editing: Daniel Joseph E. Berdida, Rizal Angelo N. Grande, Nada Alqarawi, and Ibrahim Alasqah. Visualization: Daniel Joseph E. Berdida, Rizal Angelo N. Grande. Supervision: Daniel Joseph E. Berdida and Noura Alhudaib. Project administration: Daniel Joseph E. Berdida and Noura Alhudaib. Funding acquisition: Nada Alqarawi.

## Funding

The Researchers would like to thank the Deanship of Graduate Studies and Scientific Research at Qassim University (https://www.qu.edu.sa) for financial support (QU‐APC‐2026).

## Ethics Statement

Ethics approval for this study was granted by the Ethical Research Committee of North Private College of Nursing (serial no.: NCN‐22102024‐15, approved: 24/10/2024). Permission to conduct the study was secured from the involved nursing colleges. Participants were fully explained the research aims, participant rights, and consent was voluntarily obtained. Moreover, the 2024 Declaration of Helsinki was adhered by the researchers due to involvement of human participants.

## Conflicts of Interest

Dr. Nada Alqarawi received financial support from Deanship of Graduate Studies and Scientific Research at Qassim University. This support did not influence the design, execution, or interpretation of the research. The remaining authors declare no conflicts of interest.

## Data Availability

The data that support the findings of this study are available on request from the corresponding author.
